# Biology and conservation of elasmobranchs: an introduction to the collection

**DOI:** 10.12688/f1000research.4975.1

**Published:** 2014-08-12

**Authors:** Charles W. Bangley, D. S. Shiffman

**Affiliations:** 1 Institute for Coastal Science and Policy, East Carolina University, East 5th Street, Greenville, NC, 27858; 2 Abess Center for Ecosystem Science and Policy, University of Miami, 1365 Memorial Drive, Coral Gables, FL, 33146; 3 R.J. Dunlap Marine Conservation Program, University of Miami, 4600 Rickenbacker Causeway, Miami, FL, 33149

## Abstract

Elasmobranchs, the taxonomic group comprising sharks, skates and rays, play important roles in society and marine ecology but several species in this subclass are under threat.  This collection aims to be an open access hub for articles concerning all areas of elasmobranch biology and conservation. The collection is indefinitely open to further submissions and so will continue to grow as additional articles are added.

## Editorial

There is increasing public concern about the conservation status of chondrichthyan fishes ( Simpfendorfer *et al.*, 2011). A combination of declining populations and economic importance makes management of shark, ray, and chimera fisheries a complex issue. Many conservation issues for these species are exacerbated by a lack of available scientific data ( Dulvy *et al.*, 2014) and public misunderstanding ( Neff & Heuter, 2013). The sustainability and impact of many elasmobranch fisheries, particularly in the developing world, are currently poorly understood. Even for species that are commonly caught in well-regulated fisheries and are relatively well-studied, there are still surprising discoveries being made that have implications for their conservation and management. Fortunately, researchers are developing new tools for gathering, storing, and sharing data on a global scale.

In this spirit, we are proud to present a special collection of research articles on the biology and conservation of elasmobranchs. The articles range in scope from novel observations on habitat use to the management of national-level fisheries. The tools used to answer these questions range from the very DNA of the species to databases spanning large geographical and temporal ranges. Species covered range from well-known species with decades of dedicated study to species that are only now beginning to attract research attention.

We hope that this collection will contribute to the ongoing goal of establishing sustainable shark fisheries. By making this collection open access, we hope that this information will be available to researchers, students, fishery managers, and the interested public alike.
